# Cohort study on the factors associated with survival post-cardiac arrest: Estudo de coorte dos fatores associados à sobrevivência pós-parada cardiorrespiratória

**DOI:** 10.1590/1516-3180.2015.00472607

**Published:** 2015-10-20

**Authors:** Cássia Regina Vancini-Campanharo, Rodrigo Luiz Vancini, Claudio Andre Barbosa de Lira, Marília dos Santos Andrade, Aécio Flávio Teixeira de Góis, Álvaro Nagib Atallah

**Affiliations:** I Escola Paulista de Enfermagem (EPE), Universidade Federal de São Paulo (Unifesp), São Paulo, São Paulo, Brazil.; II Sports and Physical Education Center, Universidade Federal do Espírito Santo (UFES), Vitória, Espírito Santo, Brazil.; III Human Physiology and Exercise Sector, School of Physical Education, Universidade Federal de Goiás, Goiânia, Goiás, Brazil.; IV Department of Physiology, Universidade Federal de São Paulo (USP), São Paulo, São Paulo, Brazil.; V Escola Paulista de Medicina (EPM), Universidade Federal de São Paulo (USP), São Paulo, São Paulo, Brazil.

**Keywords:** Cardiac arrest, Cardiopulmonary resuscitation, Survival analysis, Emergency medical services, Epidemiology.

## Abstract

**CONTEXT AND OBJECTIVE:**

Cardiac arrest is a common occurrence, and even with efficient emergency treatment, it is associated with a poor prognosis. Identification of predictors of survival after cardiopulmonary resuscitation may provide important information for the healthcare team and family. The aim of this study was to identify factors associated with the survival of patients treated for cardiac arrest, after a one-year follow-up period.

**DESIGN AND SETTING:**

Prospective cohort study conducted in the emergency department of a Brazilian university hospital.

**METHODS:**

The inclusion criterion was that the patients presented cardiac arrest that was treated in the emergency department (n = 285). Data were collected using the In-hospital Utstein Style template. Cox regression was used to determine which variables were associated with the survival rate (with 95% significance level).

**RESULTS:**

After one year, the survival rate was low. Among the patients treated, 39.6% experienced a return of spontaneous circulation; 18.6% survived for 24 hours and of these, 5.6% were discharged and 4.5% were alive after one year of follow-up. Patients with pulseless electrical activity were half as likely to survive as patients with ventricular fibrillation. For patients with asystole, the survival rate was 3.5 times lower than that of patients with pulseless electrical activity.

**CONCLUSIONS:**

The initial cardiac rhythm was the best predictor of patient survival. Compared with ventricular fibrillation, pulseless electrical activity was associated with shorter survival times. In turn, compared with pulseless electrical activity, asystole was associated with an even lower survival rate.

## RESUMO

**CONTEXTO E OBJETIVO:**

A parada cardiorrespiratória é comum e, mesmo com tratamentos de emergência eficientes, associa-se a prognósticos ruins. A identificação de fatores preditores de sobrevivência após ressuscitação cardiopulmonar pode fornecer informações importantes para equipe de saúde e familiares. O objetivo deste estudo foi identificar fatores associados à sobrevivência, após um ano de seguimento, de pacientes atendidos em parada cardiorrespiratória.

**DESENHO E LOCAL:**

Estudo de coorte prospectivo realizado no serviço de emergência de um hospital universitário.

**MÉTODOS:**

O critério de inclusão foi o atendimento de paciente em parada cardiorrespiratória no serviço de emergência (n = 285). Os dados foram coletados por meio do modelo In-hospital Utstein Style. Para verificar quais variáveis associaram-se à sobrevida, utilizou-se regressão de Cox (nível de significância 95%).

**RESULTADOS:**

Após um ano, a sobrevida foi baixa. Dos pacientes atendidos, 39,6% tiveram retorno da circulação espontânea, 18,6% sobreviveram até as primeiras 24 horas, sendo que, destes, 5,6% obtiveram alta hospitalar e 4,5% permaneceram vivos após um ano de seguimento. Pacientes com atividade elétrica sem pulso apresentaram duas vezes menos chances de sobreviver que aqueles com fibrilação ventricular. Nos pacientes com assistolia, as taxas de sobrevida foram 3,5 menores quando comparados aos com atividade elétrica sem pulso.

**CONCLUSÕES:**

O ritmo cardíaco inicial foi o fator preditor que melhor explicou a sobrevida. O ritmo de atividade elétrica sem pulso associou-se a menor sobrevida quando comparado a fibrilação ventricular, enquanto o ritmo de assistolia relacionou-se a ainda menor sobrevivência em relação à atividade elétrica sem pulso.

## INTRODUCTION

Several factors are associated with poor patient survival after cardiac arrest. The existence of previous cardiovascular diseases, occurrence of unwitnessed cardiac arrest, etiology, initial cardiac rhythm, ineffective cardiopulmonary resuscitation, prolonged cardiopulmonary resuscitation, lack of ongoing training of healthcare staff in cardiopulmonary resuscitation techniques, delays in defibrillation, inadequate emergency care structure and lack of adherence to care protocols for cardiac arrest by healthcare services may all have a crucial bearing on patient survival.^1^ ^2^

In the United States, over 300,000 individuals are admitted to emergency departments each year due to cardiac arrest.^3^ ^4^ In Brazil, approximately 200,000 cases of cardiac arrest occur each year, of which half occur within the hospital environment. The return of spontaneous circulation, i.e. the presence of any palpable central pulse during cardiopulmonary resuscitation, is decisive for patient survival and may be obtained in 50% of cases.^1^ ^3^

The majority of deaths occur during the first 24 hours after cardiac arrest, and the rate of survival until hospital discharge ranges from 9.5% (in-hospital cardiac arrest) to 24.2% (extrahospital cardiac arrest).^3^ In most cases, survival can be predicted from the heart and brain damage caused by ischemia followed by reperfusion. Fifty percent of survivors retain irreversible neurological sequelae, with losses of functional ability, cognition, emotional wellbeing and quality of life.^3^

Despite the existence of guidelines for treating cardiac arrest, the survival rates and prognoses are highly variable, primarily due to the heterogeneity of the population served (age, personal history and pre-cardiac arrest neurological status).^3^ Greater understanding of factors associated with survival after cardiac arrest in a highcomplexity-care referral hospital can therefore help in developing and proposing preventative strategies and in optimizing resources (physical, human and material) for cardiac arrest care, which may improve patient survival and prognosis.

## OBJECTIVE

To identify factors associated with the survival rate among cardiac arrest patients treated in the emergency department of a university hospital during the first 24 hours, at discharge and six months and one year after cardiac arrest.

## METHODS

This study was a prospective and descriptive cohort study on patients treated for cardiac arrest in the emergency department of the Federal Teaching Hospital in São Paulo, Brazil.

This hospital is one of the largest federal teaching hospitals in Brazil, serving an area that contains more than five million inhabitants and performing high-complexity procedures. Each month, the hospital conducts more than 90,000 medical appointments, 2,600 admissions, 1,600 operations and approximately 290,000 laboratory tests. Each day, approximately 4,000 patients are treated in outpatient clinics, and 1,000 are treated in the emergency department. The care team consists of more than 840 residents; approximately 12,000 undergraduate, postgraduate, and specialization students; and more than 5,300 employees, including teachers, professionals in various areas of healthcare and administrative workers.^5^

### Patients

The sample was obtained successively and consisted of 285 patients who were diagnosed with cardiac arrest. The patients were selected consecutively during the period from February 1, 2011, to January 31, 2012, and were followed for one year after enrolment in the study. There was no loss from follow-up.

The inclusion criterion was that the patients presented cardiac arrest that occurred in an extra-hospital or in-hospital environment and were treated in the clinical emergency department. The patients were selected after admission to the emergency department, and significant data relating to the cardiac arrest were gathered in outpatient cases from family members and from the team of nursing professionals (pre-hospital care). Individuals presenting cardiac arrest who were treated in other hospital departments were excluded from the study.

### Data collection

Data collection was performed by trained nurses and was monitored by the study's lead researcher via daily calls during all shifts and through visits to the emergency department. For data collection, the In-hospital Utstein Style^6^ template was used. This consists of a standard report that is used to collect meaningful data on cardiac arrest and cardiopulmonary resuscitation maneuvers, and information relating to the patient and the outcomes presented at hospital discharge, six months later and after one year of follow-up.

The data were collected in five steps. In the first step, during cardiac arrest, the following information was collected: gender, age, skin color, location of cardiac arrest occurrence, whether the cardiac arrest was witnessed, presumed immediate cause of the arrest, initial cardiac rhythm, whether cardiopulmonary resuscitation was attempted, basic and advanced life support maneuvers that were performed during care, interval between the start of cardiopulmonary resuscitation and first defibrillation attempt, interval between the start of cardiopulmonary resuscitation and performing tracheal intubation, interval between the start of cardiopulmonary resuscitation and the first dose of epinephrine, interval between the start and end of cardiopulmonary resuscitation and the occurrence of the return of spontaneous circulation. In the second and third steps, data were collected 24 hours after the return of spontaneous circulation and at hospital discharge, respectively. In the fourth and fifth steps, data were collected six months and one year after hospital discharge, respectively. The outcomes examined in these steps included patient survival and the neurological status of the survivors.

The neurological status of the survivors was assessed using the Glasgow-Pittsburgh Cerebral Performance Category scale during visits to inpatient units and through phone calls and outpatient appointments after hospital discharge. The Cerebral Performance Category scale is divided into five categories. Category 1 indicates complete independence and the ability to work; Category 2 indicates moderate disability, the ability to work part-time and independence in activities of daily living; Category 3 indicates severe disability and total dependence in activities of daily living; Category 4 indicates a persistent vegetative state; and Category 5 indicates brain death.^6^

**Figure 1:**
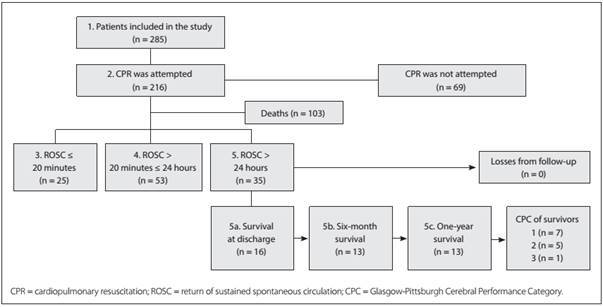
Flowchart of patient follow-up in the study.

This study was approved by the Research Ethics Committee of Universidade Federal de São Paulo (protocol number: 0030/2011). All procedures were performed in accordance with the Declaration of Helsinki. Due to the observational nature of the data collection and the severity of the patients' conditions, the study was granted a release from acquisition of informed consent. Figure 1 presents the flowchart of patient follow-up.

### Statistical analysis

The statistical analysis was performed by means of SPSS version 19 (Chicago, IL, USA). Data are presented using descriptive statistics. The mean, standard deviation, median and minimum and maximum values were calculated for continuous variables. The frequency and percentage were calculated for categorical variables. If a patient experienced more than one episode of cardiac arrest, the statistical analysis was performed considering only the first event, because the inferences were made regarding individuals' variables and not those of the cardiac arrest.

The continuous variables included age, number of days of hospitalization, time from the start of cardiopulmonary resuscitation until the first defibrillation attempt, time from the start of cardiopulmonary resuscitation until tracheal intubation, time from the start of cardiopulmonary resuscitation until the first dose of epinephrine and the duration of cardiopulmonary resuscitation. These variables were categorized for use in the survival analyses. Categorizations were made using the mean of the results.

Survival curves for the first 24 hours after cardiopulmonary resuscitation, until hospital discharge and six months and one year after hospital discharge were estimated using the KaplanMeier method, taking the patient's death to be the event.

Cox regression was used to verify the factors that influenced patient survival. Simple regression was initially performed to analyze the relationship between the dependent variable, in this case the occurrence of death, and each independent variable individually. A multiple regression model was then produced, taking all the independent variables into consideration. To form the sample for this, we took into account the patients for whom information on all the variables used in the model was available. Thus, patients for whom information on at least one of the variables was missing were excluded from the analysis, using the forward method. The final sample for this totaled 217 patients. The variables considered in the regression model consisted of age, gender, skin color, personal history, presence of consciousness, breathing and pulse on admission, pre-cardiac arrest Cerebral Performance Category score, occurrence of previous cardiac arrest, location of cardiac arrest, whether the cardiac arrest was witnessed, presumed immediate cause of cardiac arrest and initial rhythm of cardiac arrest.

Significant associations between variables were calculated using the hazard ratio, which takes into consideration the time for which each patient was followed up. The level of statistical significance was 0.05.

## RESULTS

The median patient age was 68 years (range: 17-101) (n = 285), with predominance of males (55.8%, n = 159) and white patients (71.9%, n = 205). On admission, 63.8% (n = 182) of the patients were conscious, 69.8% (n = 199) were breathing and 76.5% (n = 218) had a pulse. 

With regard to the characteristics of cardiac arrest, most cases occurred in the hospital environment (76.5%, n = 218), and 88.7% (n = 253) of them were witnessed by a healthcare professional or family member. The immediate cause of cardiac arrest was identified in 93.3% (n = 266) of the cases; the most frequent cause was respiratory failure (30.8%, n = 82), and pulseless electrical activity was the most common initial cardiac rhythm (48.4%, n = 138). In 75.8% of the patients (n = 216), cardiopulmonary resuscitation was attempted by means of ventilation, and external chest compressions were performed. Defibrillation was required in 20.3% (n = 44) of the subjects, 51.9% (n = 148) underwent tracheal intubation, and 73.7% (n = 210) received epinephrine (Table 1).

The median time from the start of cardiopulmonary resuscitation until the first defibrillation attempt was five minutes (range: 0-55), the time until completion of intubation was four minutes (range: 0-48), and the time until the first dose of epinephrine was one minute (range: 0-33). The median duration of cardiopulmonary resuscitation was 17 minutes (range: 2-76).

**Table 1:**
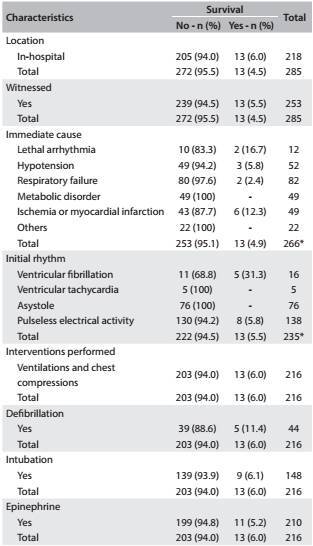
Characteristics of the cardiac arrest episodes of the study population.

*Total number of patients with this information available.

### Outcomes from cardiac arrest treatment

Among all the patients (n = 285), 39.6% (n = 113) presented return of spontaneous circulation; 18.6% (n = 53) survived until 24 hours after the cardiac arrest; 5.6% (n = 16) were discharged; and only 4.5% (n = 13) were alive after one year of follow-up.

### Survival analysis using the Cox regression model

The dependent variable for Cox regression model was taken to be the occurrence of death and the independent variables considered were: age, gender, skin color, personal history, presence of consciousness, breathing and pulse on admission, pre-cardiac arrest Cerebral Performance Category score, occurrence of previous cardiac arrest, location of cardiac arrest, whether the cardiac arrest was witnessed, presumed immediate cause of cardiac arrest and initial rhythm of cardiac arrest.

Multiple regression analysis revealed that the clinical variable most strongly associated with patient survival was the initial cardiac rhythm. Over the first 24 hours after cardiac arrest (P = 0.0009) and at discharge (P = 0.0004), asystole, compared with pulseless electrical activity, was associated with a lower survival rate. Over the first six months after hospital discharge, pulseless electrical activity, compared with ventricular fibrillation, was associated with a lower survival rate (P = 0.0371); in turn, asystole, compared with pulseless electrical activity, was related to an even lower chance of survival (P = 0.0003). One year after hospital discharge, pulseless electrical activity, compared with ventricular tachycardia, was associated with a lower chance of survival (P ≤ 0.0001); in turn, asystole, compared with pulseless electrical activity, was related to an even lower survival rate (P = 0.0310) (Table 2).

**Table 2:**
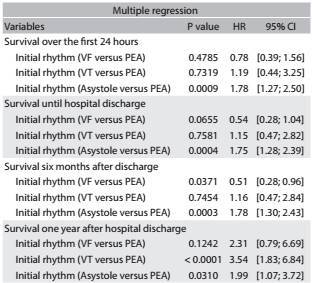
Factors associated with survival over the first 24 hours after cardiac arrest, at hospital discharge and at six months and one year after discharge.

HR = hazard ratio; CI = confience interval; VF = ventricular firillation; PEA = pulseless electrical activity; VT = ventricular tachycardia. Statistically signifiant diffrences were noted for P values ≤ 0.05.

## DISCUSSION

Cardiac arrest occurs frequently and is potentially fatal.^7^ The recommended treatment for individuals presenting cardiac arrest is based on the chain of survival, with emphasis on the quality of external chest compressions, early defibrillation and implementation of a post-cardiac arrest care protocol, because several physiological systems are affected during cardiac arrest and the majority of patients with a return of spontaneous circulation do not survive the first 24 hours.^8^ Survival rates until hospital discharge range from 9.5 to 24%, due to the severity of the clinical conditions presented by patients, which include high morbidity and mortality rates and neurological sequelae.^7^ ^9^ ^11^

In this study, among all the patients (n = 285), 39.6% presented return of spontaneous circulation, 18.6% (n = 53) survived until 24 hours after cardiac arrest, 5.6% (n = 16) were discharged and only 4.5% (n = 13) were alive after one year of follow-up. The variable that best explained the survival rate after one year was the initial cardiac rhythm.

### Survival over the first 24 hours after cardiac arrest and at discharge

The determining factors for survival over the first 24 hours are related to the complexity of the condition of the patient presenting cardiac arrest, and to implementation of immediate cardiopulmonary resuscitation, early defibrillation and a post-cardiac arrest care plan with the goals of improving cardiopulmonary and brain function and correcting metabolic abnormalities.^8^ In this study, in the majority of the cases (48.4%), the initial rhythm comprised pulseless electrical activity, which can be explained by the fact that most cardiac arrest cases occurred in an in-hospital environment, in high-severity patients.^12^ This may explain the low rates of return of spontaneous circulation (39.6%) and long-term survival (4.5%). Moreover, the initial rhythm was the parameter most strongly associated with the patient survival rate during the first 24 hours after cardiac arrest (P = 0.0009) and at discharge (P = 0.0004). In patients with asystole as the initial cardiac rhythm, the rate of survival was lower than that for patients with pulseless electrical activity.

A study conducted to evaluate improvements in the survival of patients who suffered in-hospital cardiac arrest showed that there was a significantly higher hospital survival rate among patients with a shockable initial cardiac rhythm (ventricular fibrillation and ventricular tachycardia; odds ratio 0.4; 95% confidence interval: 0.2-0.6; P = 0.001).^12^ Our findings are similar to the findings reported in the literature, since the low survival rate found over the first 24 hours after cardiac arrest (18.6%) can be explained by the fact that the majority of the patients treated (75.0%) presented asystole or pulseless electrical activity as their initial cardiac rhythm.

The initial cardiac rhythm found most commonly in adults is ventricular fibrillation, i.e. a chaotic, disorganized rhythm incapable of generating circulation. If this is not promptly treated with defibrillation, it may progress to asystole.^13^ In such cases, immediate cardiopulmonary resuscitation and early defibrillation are determinants for the survival of these patients.^13^ ^14^

Ventricular fibrillation is the initial cardiac rhythm associated with greatest likelihood of a return to spontaneous circulation, provided that cardiopulmonary resuscitation and early defibrillation are performed.^3^ ^4^ The execution of defibrillation during cardiopulmonary resuscitation maneuvers is based on cardiac arrest time phases; during the electrical phase (three to four minutes), immediate defibrillation is recommended. The time between cardiovascular collapse and performing the first defibrillation attempt are important determinants of better survival rates.^3^ ^4^ ^9^ ^15^ ^16^ The literature suggests that use of an automated external defibrillator in in-hospital cardiac arrest situations can reduce the time until performing the first defibrillation attempt, thereby improving survival.^17^ ^18^ In a meta-analysis involving 142,740 patients who suffered extra-hospital cardiac arrest and had an initial rhythm of ventricular fibrillation or ventricular tachycardia, a greater chance of a return to spontaneous circulation was found, depending on the location of the occurrence of the event (hazard ratio = 7.6). This finding highlights the need for automated external defibrillators to be made available in public places and for training to be provided for the population with regard to identification of cardiac arrest and performing of cardiopulmonary resuscitation and early defibrillation.^19^ In Brazil, it has been shown that patients with ventricular fibrillation or ventricular tachycardia as the initial cardiac rhythm have a higher probability of a return to spontaneous circulation and survival until hospital discharge (35.0%) than do patients with other heart rhythms (4.0%).^2^ ^20^

### Survival at six months and one year after hospital discharge

Little is known regarding the rates of long-term survival after cardiac arrest. In addition, the few studies that have been conducted^21^ ^22^ have had limited sample sizes, which impairs possible comparisons between samples. Given that most patients do not survive until one year after the event, usually as a result of persistent brain damage, obtaining a reliable overall estimate of long-term survival and the factors associated with it could improve the practice of cardiopulmonary resuscitation.^22^ In our study, long-term survival was classified as a period of six months to one year.^21^ ^22^ Over the first six months after hospital discharge, pulseless electrical activity was associated with a lower survival rate than ventricular fibrillation (P = 0.0371), and asystole was related to a lower chance of survival compared with pulseless electrical activity (P = 0.0003). One year after hospital discharge, the survival rate of patients with pulseless electrical activity was lower than that of patients with ventricular tachycardia (P ≤ 0.0001), and the survival rate of patients with asystole was lower than that of patients with pulseless electrical activity as the initial cardiac rhythm (P = 0.0310).

The evidence suggests that the overall incidence of cardiac arrest and patient survival rates are quite heterogeneous. Moreover, it has been observed that the rate of occurrence of cardiac arrest with a shockable initial rhythm (ventricular fibrillation and ventricular tachycardia) has decreased, which may be attributed to the use of beta-blockers in prevention of coronary disease and to implantation of cardioverter-defibrillators. Better understanding of the variability of this event is critical for prevention of cardiac arrest and implementation of effective cardiopulmonary resuscitation practices.^23^ ^25^

The strengths of this study include its prospective data collection from consecutive patients and follow-up of these patients for one year with no losses. Another point was that the work was conducted in a referral university hospital. One limitation of the study is its lack of information on care in the pre-hospital environment, including a lack of data regarding the influence of the time from the collapse until the beginning of cardiopulmonary maneuvers, on the survival of patients in cases of extra-hospital cardiac arrest. No additional data from the time after discharge was available, such as whether patients were included in rehabilitation programs.

The main cause of cardiac arrests in adults is ventricular fibrillation, which tends to evolve into asystole within only a few minutes. Therefore, in characterizing groups at high risk of cardiac arrest, implementation of an integrated registration system for cardiac arrest data and strategies for early detection and treatment, such as training and education of the lay population and healthcare professionals on cardiopulmonary resuscitation maneuvers and on the use of automated external defibrillators, is essential and can increase patient survival.

## CONCLUSIONS

In conclusion, the long-term survival after cardiac arrest was low in this study. The variable that best explained the survival rate was the initial cardiac rhythm. Over the first 24 hours after cardiac arrest and at discharge, asystole, compared with pulseless electrical activity, was associated with a lower survival rate. Six months after discharge, pulseless electrical activity, compared with ventricular fibrillation, was associated with a lower survival rate; in turn, asystole, compared with pulseless electrical activity, was related to an even lower chance of survival. After one year of follow-up, pulseless electrical activity, compared with ventricular tachycardia, was associated with a lower chance of survival; in turn, asystole, compared with pulseless electrical activity, was related to an even lower survival rate.
